# Flexing with lignin: lignin-based elastomers synthesised from untreated kraft black liquor†

**DOI:** 10.1039/d4py00490f

**Published:** 2024-06-03

**Authors:** Philip Verdross, Robert T. Woodward, Alexander Bismarck

**Affiliations:** a Polymer and Composite Engineering (PaCE) Group, Institute of Material Chemistry and Research, Faculty of Chemistry, University of Vienna Waehringer Strasse 42 1090 Vienna Austria robert.woodward@univie.ac.at alexander.bismarck@univie.ac.at; b Department of Chemical Engineering, Imperial College London South Kensington Campus London SW7 2AZ UK

## Abstract

The synthesis and characterisation of a lignin-based elastomer system using lignin-epoxy-resins is presented. Untreated kraft black liquor was used to synthesise glycidyl lignin or black liquor-based epoxy resin (BLER), following a published procedure. A flexible, elastomeric thermoset was produced by cross-linking BLER with succinic anhydride (SA). The produced material was characterised in respect to its chemical, thermal, mechanical and swelling characteristics. In addition, vertical burning tests were performed. The obtained lignin-based elastomeric thermoset had a tensile strength of 1.0 ± 0.20 MPa and elastic moduli of 1.6 ± 1.4 and 0.44 ± 0.35 MPa at 5% and 50% elongation, respectively. A maximum elongation of 151 ± 49% was found.

## Abstract 2

The synthesis and characterisation of a lignin-based elastomer system using lignin-epoxy-resins is presented.

## Graphical abstract

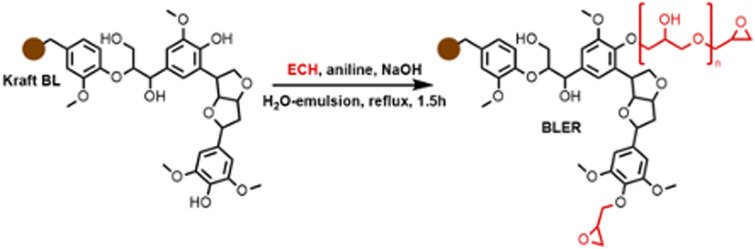

## Introduction

Lignin, as a by-product of the pulp and paper industry,^1^ is a naturally occurring and renewable resource that is produced on a scale of millions of metric tons per year.^2^ Lignin is used almost exclusively as a fuel to generate energy for the pulping process. Lignin is an abundant source of biogenic carbon that could substitute fossil carbon sources in applications such as fuel production,^3^ production of organic small molecules^4,5^ or the synthesis of polymeric precursors^6^ and materials.^7^ Especially interesting in the context of lignin valorisation and upcycling is black liquor (BL), since it can be collected directly from the pulping process without further refining. BL is a complex solution of organic and inorganic components, comprising lignin, tall oils and polysaccharides that were separated from plant mass or wood during pulping.^8,9^ While lignin can be precipitated under acidic conditions from BL,^10,11^ producing hydrogen sulfide in the process, it is more appealing to avoid the consumption of chemical resources while still producing valuable products from BL confirmed by life cycle assessment.^12^

A variety of approaches have been pursued for the direct use of BL in the production of polymeric materials or graphenes.^13^ Examples include copolymerization of lignin with *N*,*N*′-methylenebis-acrylamide,^14^ the frothing of BL with epichlorohydrin (ECH)^15^ to yield lignin based foams, or the production of carbide based materials.^16^ Rubbers from refined lignin have been developed using epoxide-based approaches, for example using diglycidyl ether polyethylene glycol^17^ or Novolac systems.^18^ Mixtures of polyurethanes with lignin have also been shown to yield elastomeric materials.^19^ In regard of rubbery materials in general, lignin is often used as a filler.^20^ Toughening of natural rubber^21^ can be achieved using lignin, representing an interesting application, because of the combination of different natural resources in a material. Lignin was also found to have positive effects on the mechanical properties of natural rubber and its resistance to oxidation.^22^

While approaches that use purified lignin can yield usable materials, in an ideal case the isolation of lignin from BL should be avoided due to unnecessary consumption of energy and resources. A liquid precursor to a thermosetting system is desirable. Lignins in general are solids at room temperature, the impregnation of fabrics can represent a challenging step during materials fabrication, for instance for truck tarpaulins, rubber boots and rain jackets, or synthetic leathers. We reported the synthesis of liquid epoxy resins from BL directly, *i.e.* without prior purification of the lignin.^23^

Building from our previous findings on the production of thermosets from BL, we hypothesised that the use of a hardener with a flexible backbone may yield rubbers from black liquor epoxy resins (BLER). Herein, succinic anhydride (SA) was chosen as a crosslinker with a flexible aliphatic backbone for the production of rubbers from BLER due to its possible production from biomass,^24^ or from lignin^25^ or carbohydrates in an ecologically sustainable way.^26^ By screening anhydrides that do not have a flexible backbone, we found that only SA leads to elastomeric materials. We characterised BLER-SA thermosets in regard of key-properties of elastomers, such as mechanical properties, thermal stability, swelling in solvents or resistance to solvents and gel content.

Considering that only about 2% of the lignin that is produced annually is used in any upcycling processes^27,28^ besides use as fuel, it is an attractive platform to create a multitude of materials that could help to substitute oil in the production of polymers.

## Materials and methods

### Chemical compounds

Magnesium sulfate, sodium hydroxide, ethanol and succinic anhydride (SA) were purchased from Sigma Aldrich. Aniline (96%), oxophthalic anhydride, phthalic anhydride, 4,4′-carbonyldiphthalic anhydride and epichlorohydrin (ECH) were bought from Tokyo Chemical Industries (TCI). Dichloromethane, toluene, and chloroform were bought from VWR Germany. Black liquor (BL) was provided by Zellstoff AG Poels (60 wt% dry mass).

### Synthesis of BLER resin

BLER was synthesized as described previously (detailed chemical information about starting materials and prepolymers is given; the same black liquor has been used).^23^ 300 mL of BL, 300 mL of ECH and 6.5 mL of aniline were heated to reflux and stirred mechanically. A solution of 45 g sodium hydroxide in 200 mL of distilled water was added over 30 min using a dropping funnel. The solution was stirred under reflux for an additional 1.5 h. The mixture was extracted using three 250 mL portions of DCM. The combined organic phase was dried using magnesium sulfate. Magnesium sulfate and solid particles were filter off and organic volatiles were removed using a rotary evaporator at reduced pressure. All the DCM and 7 mL of ECH were recovered during this step. The obtained viscous product was dried in a vacuum oven at 120 °C and 10 mbar until no further loss of mass could be observed (1.5 h).

### Polymerization method

Synthesized BLER was mixed directly with SA. A ratio of three parts resin to one part hardener by weight was used, corresponding to a ratio of anhydride-functionality per gram resin of 3.3 × 10^−3^ mol g^−1^. The mixture of powdered SA and liquid BLER was heated to 60 °C–80 °C using a heat gun for 1 minute while stirring manually. SA dissolves completely in the resin during heating. The homogeneous resin-hardener-mixture was poured into molds and cured in an oven at 140 °C for 24 h and post treated at 70 °C for 1 h. The epoxy equivalent weight of BLER was titrated according to literature.^29^ It was found to be 850 g mol^−1^. To screen anhydrides other than SA as curing agents, oxophthalic anhydride, phthalic anhydride and 4,4′-carbonyldiphthalic anhydride were used. They were added as solids/powders to BLER and stirred at 60°–80 °C until they completely dissolved in the liquid resin. They were all added in the same ratio of functional groups to resin as SA and cured according to the procedure used for BLER and SA. Samples were cured in an open silicon mold (ice cube form), so that little blocks (2 × 2 × 0.5 cm) or lumps were obtained for visual and tactile inspection.

### Isolation of hemicellulose

Aqueous phase (15 mL) obtained upon extraction of the reaction mixture with DCM was diluted in an excess of acetone. A white precipitate formed and was filtered from the liquid and dried at 95 °C and 25 mbar until constant mass was obtained (2 h). The mass of the dried precipitate was 1.1 mg.

### Chemical constitution and progress of cure *via* infrared spectroscopy

All spectra were recorded using a Bruker Platinum II spectrometer and the OPUS software package from Bruker. Scans were recorded within the wavenumber range 400–4000 cm^−1^ with a resolution of 4 cm^−1^. 32 scans were recorded and averaged per spectrum. Liquid samples were investigated as thin films.

### Thermal and calorimetric analysis

All differential scanning thermograms were recorded using a TA instruments discovery series differential scanning calorimeter. Samples were placed into non-hermetic T-zero aluminum crucibles. All data was recorded under a constant nitrogen flow of 5 mL min^−1^. A heating rate of 5 °C min^−1^ was used to record heat flow during the curing process and the glass transition temperature *T*_g_. Heating rates of 5 °C, 10 °C, 15 °C and 20 °C min^−1^ were used to estimate the activation energy of polymerization reactions by using the Kissinger method.^30^ Data collection and analysis were done using the accompanying TA-DSC software.

Decomposition behavior of samples was monitored by thermogravimetric analysis using a TGA 5500 (TA instruments). Cured samples were heated from 50 °C to 800 °C with a heating rate of 10 °C min^−1^. Measurements were performed in a constant gas flow using air with a flow rate of 10 mL min^−1^. Platinum crucibles were used.

### Swelling tests and determination of gel content

The following procedures were adapted according to published literature for other lignin thermosets.^31^ To investigate swelling, samples of an approximate mass of 0.2 g and dimensions of 10 × 10 × 1 mm were immersed in 10 mL of water, toluene, ethanol, or chloroform. Sealed glass vessels were used to avoid evaporation. Samples were cured as indicated in the section “Polymerization method”. Samples were kept immersed in solvent for one, five and ten days, and then were dried using tissue and their masses were recorded. The swelling capacity *S* [wt%] was calculated according to eqn (1):1
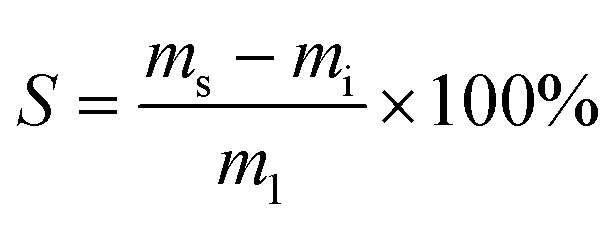
with *m*_i_ being the initial mass of sample and *m*_s_ the mass of swollen sample.

The gel content GC [wt%] was determined by extracting samples for 10 days with ethanol. After the treatment, specimens were removed from the solvents and dried using tissue. After that, solvent was removed at 140 °C and 10 mbar until constant weight was recorded. We calculated GCs using eqn (2):2
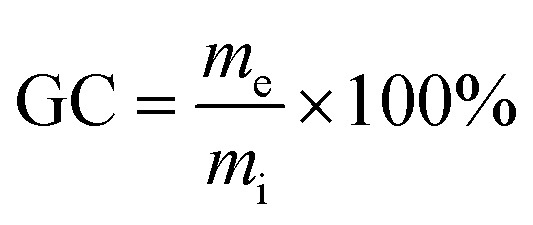
where *m*_e_ is the mass of specimens after extraction and *m*_i_ is the mass of initial specimens before swelling.

### Mechanical testing

Tensile tests were carried out according to ASTM D638 using a 1 kN load cell (Instron 5969 universal dual-beam testing device). Specimens were produced by curing BLER-SA in a mould as a flat sheet and stamping out dumbbell-shaped specimens of 1.6 mm thickness, 5.0 mm width, and a gauge length of 50 mm. Tests were carried out at a rate of 50 mm min^−1^ at 21 °C and 35% relative humidity (RH). Specemines were pre-conditioned for one week under these conditions before testing. Specimens prepared in the same way were tested using cyclic mechanical testing and the same instrumentation was used. During cyclic testing, specimens were subjected to elongations of 5, 10, 20, 30 and 40 mm, consecutively. Each cycle comprised elongation at a rate of 8.3 mm s^−1^, a 10 s hold, and then relaxation to an extension of 0 mm, again at a rate of 8.3 mm s^−1^, followed by another hold of 10 s. All tests were performed at 21 °C and 35% RH. Six specimens were tested. All specimens were conditioned at the relative humidity and testing temperature for 48 h prior to mechanical testing.

Shore hardness was tested using a type A Shore durometer (ASTM 2240). All specimens were conditioned at the relative humidity and testing temperature for 48 h prior to hardness testing. Indentation tests were done on films of 1.6 mm thickness (1 × 1 cm), cut out from a bigger sheet of film. Five specimens were tested at 21 °C and 35% RH. Values were recorded after 3 and 15 s, respectively.

### Vertical burning tests

For vertical burning tests, specimens 1 mm thick, 50 mm long, and 5 mm in width were used. Specimens were cured as described above. The flame of a Bunsen burner was applied to the bottom of each specimen for 10 s and then removed. The time until full flame extinction and the time of afterglow were recorded. Specimens were then again subjected to the flame of a Bunsen burner for 10 s and time until flame extinction (if ignited) and time of afterglow were again recorded. The difference in mass before and after ignition was recorded. The height of the flame was approximately 20 mm for every test. We recorded individual impressions such as the smell and character of the evolving smoke and flame.

### Surface morphology upon cure

Scanning electron microscopy (SEM, JMC-6000 SEM, JEOL) was used to image the surface of different specimens. All specimens were placed on conductive tape and sputter-coated with a thin film of gold (JFC 1200 Fine Coater, JEOL) at a current of 30 mA for 30 s, prior to imaging. SE micrographs were recorded at an acceleration voltage of 15 kV. To investigate cured samples surfaces, unbroken samples were cleaned with isopropanol and then sputtered after drying.

## Results and discussion

### Chemical and thermal analysis of lignin-based elastomers

**Scheme 1:**
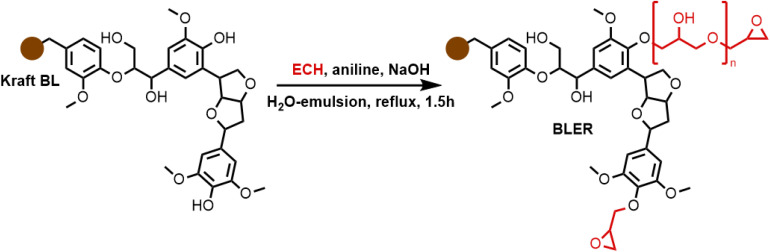
The formation of BLER by reacting ECH with lignin contained in raw black liquor.

The formation of BLER, as described previously,^32^ (Scheme 1) proceeds *via* the grafting of glycidyl ethers onto lignin. Phenols contained in lignin are deprotonated under alkaline conditions and react with ECH, catalysed by aniline that forms quaternary ammonium salts and higher substituted amines that help the formation of glycidyl ethers or polyglycidyl ethers (Scheme 1 in red). The reaction mixture was then extracted with DCM and hemicelluloses, as well as inorganic components, were retained in the aqueous phase of the extraction. Hemicelluloses were precipitated in an excess of acetone. An FT-IR spectrum of hemicelluloses recovered from the aqueous phase is provided in the ESI† and was compared to literature for identification.^33^ Extensive analysis of BL used in the present study and BLER is provided in our previous publication.^32^

**Fig. 1:**
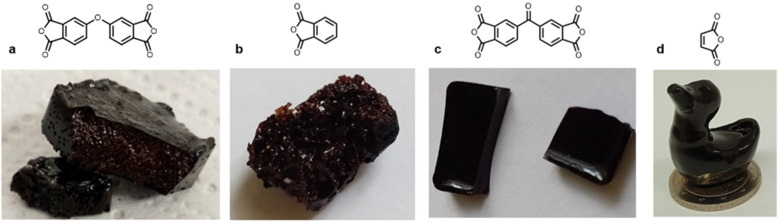
Different thermosets obtained by reacting BLER with (a) oxophthalic anhydride, (b) phthalic anhydride, (c) 4,4′-carbonyldiphthalic anhydride, and (d) maleic anhydride (cured according to previously published procedure^23^).

In our previous work, maleic anhydride was used to cure BLER, yielding a stiff thermoset. Here, we target an elastomeric compound and so employ succinic anhydride (SA) as both a flexible aliphatic anhydride backbone, as well as an acid anhydride that can be bio-sourced.^25,26,34^ In preliminary tests with aromatic anhydrides having no flexible backbone (Fig. 1), we formed stiff or brittle thermosets. We chose aromatic anhydrides due to their solubility in BLER and non-flexible or partially flexible structure. Only 4,4′-carbonyldiphthalic anhydride yielded smooth and bubble-free thermosets, however, they were not flexible materials. The other tested anhydrides could not be cured to obtain samples that were neither flexible nor free of severe defects. The bubbles formed during curing were ascribed to decarboxylation of the curing agent, forming CO_2_ leading to gas enclosures forming voids in the cured polymer. Due to the undesirable mechanical properties of the formulations, paired with a lack of bio-sourceability, no further tests were carried out.

**Fig. 2:**
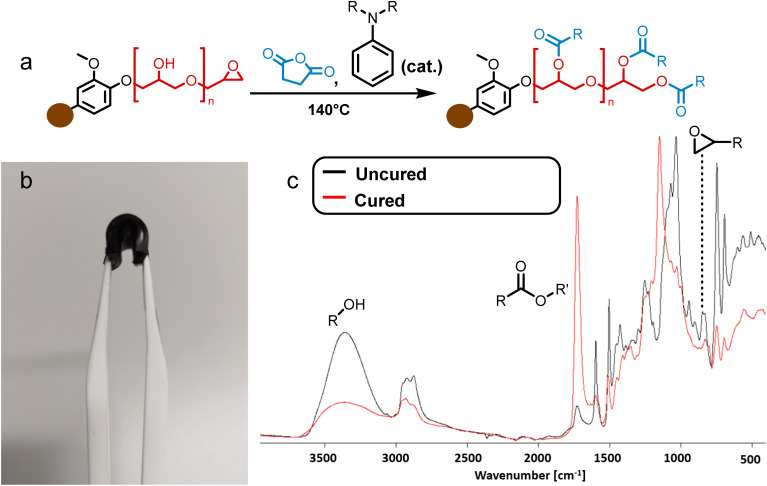
(a) Reactions that lead to the formation of BLER-SA by cross-linking of glycidyl ethers/alcohols and SA. (b) A piece of cured BLER bent with tweezers. (c) FT-IR spectra of uncured BLER (black) and BLER that was cured with SA (red) with marked bands for epoxides, esters and hydroxy groups.

To produce elastomers, BLER was mixed with SA and cured at elevated temperature in an oven. The completion of the reaction was confirmed *via* FT-IR (Fig. 2c) by observing bands attributed to epoxides at 822 cm^−1^. Upon curing, a pronounced band at 1732 cm^−1^ indicated the formation of esters, due to reactions between the anhydride hardener and the glycidyl groups, as well as hydroxy groups present in BLER. Signals related to hydroxy groups at 3350 cm^−1^ were less pronounced upon curing due to esterification. It is also plausible that unreacted carboxylic acid groups contribute to the broad band at 3350 cm^−1^. A band in the region of ether bonds (1100 cm^−1^) was also shifted, probably due to the cross-linking of glycidyl ethers by SA. The material is black and opaque after curing, as well as glossy. In general, the materials retained their moulded shape.

**Fig. 3:**
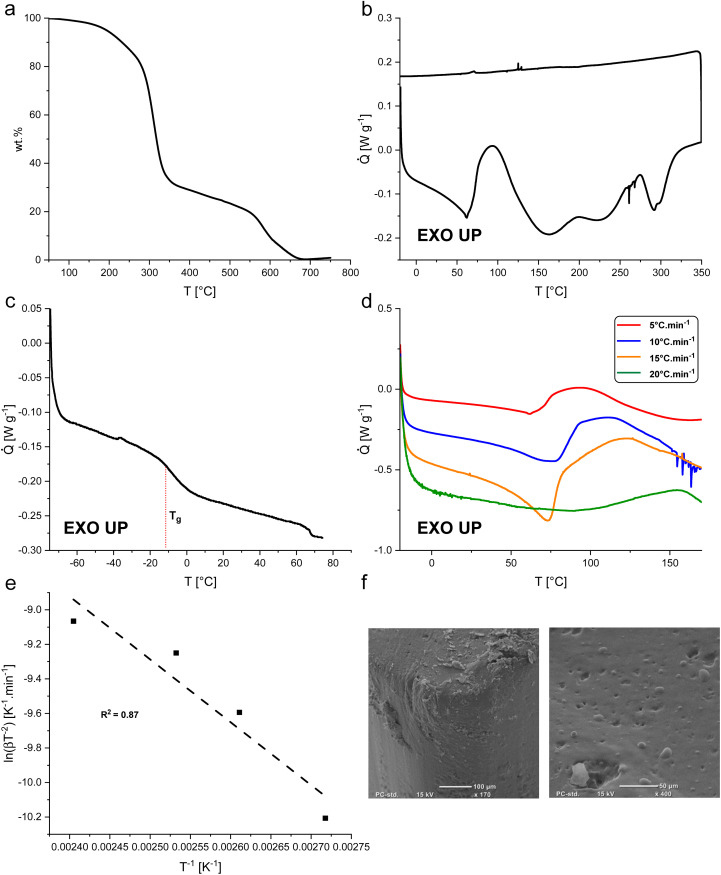
(a) Thermogravimetric data from decomposition of BLER-SA in air. (b) Thermogram of the curing of BLER-SA under non-isothermal conditions comprising exotherms of cure as well as decomposition. (c) Non-isothermal thermogram of cured BLER-SA at low temperatures showing the glass transition temperature *T*_g_. (d) Thermograms of BLER-SA curing at different heating rates to determine the activation energy of curing. Heating rates of 5 °C min^−1^ (red curve), 10 °C min^−1^ (blue curve), 15 °C min^−1^ (orange curve), and 20 °C min^−1^ (green curve) were recorded. (e) Kissinger plot of BLER-SA curing with given squared residuals (*R*^2^ = 0.87). (f) Micrographs of cured surfaces of BLER-SA (surfaces facing the mould).

BLER-SA begins to decompose in air at about 200 °C, as determined by TGA (Fig. 3a). In the first decomposition process, approximately 70% of BLER-SA's mass is lost. In a second decomposition process that occurs above 550 °C, further mass is lost until a residual mass of 0.3 wt%, attributed to minerals from ash contained in BL. The first decomposition process is attributed to the degradation of aliphatic moieties in the polymer structure, while the second decomposition is attributed to the degradation of its aromatic components.^35^

The curing of BLER with SA proceeds at temperatures above 60 °C, as determined by DSC (Fig. 3b). Four distinct exotherms were observed in the temperature range of 20°–350 °C. The first exotherm was attributed to crosslinking of epoxy-moieties and potential ester formation between SA and hydroxy groups in BLER, while further exotherms were attributed to decomposition by comparison of results from TGA. A decrease of the heat flow before curing was attributed to the dissolution of previously undissolved or recrystallised SA. After full cure, the material has a glass transition temperature *T*_g_ of −6 °C, as determined by DSC at a heating rate of 5 °C min^−1^. The enthalpy-change during the first exotherm is 99 J g^−1^. The activation energy of the curing reactions was determined by the Kissinger method,^30^ recording thermograms at different temperature ramps and determining the corresponding peak temperatures. By plotting the inverse peak temperatures against the natural logarithm of the heating rates and the corresponding inverse square of the peak temperatures, a linear trend can be observed (Fig. 3e). The activation energy can be deduced from the slope inherent to the linear regression (eqn (3)):3
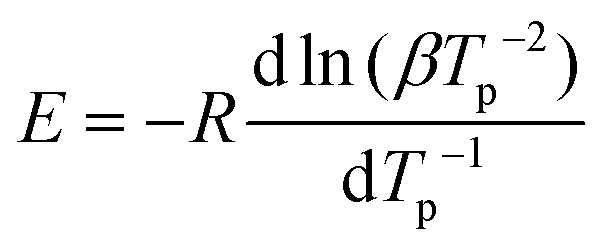
where *T*_p_ denotes a corresponding peak temperature, *β* a heating rate, *E* the activation energy and *R* the universal gas constant. Using eqn (3), the activation energy of the first exotherm was deduced to be 30 kJ mol^−1^. A higher activation energy would be expected for the curing of epoxy resins with carboxylic acid anhydrides, ranging from approximately 40–100 kJ mol^−1^, even if an accelerator (tertiary amine) is added.^36^ The relatively low activation energy was attributed to the presence of catalyst from the synthesis of BLER (determined by NMR spectroscopy).^23^ Aniline is deployed in a one-pot approach together with black liquor and ECH, so that the nitrogen moiety of aniline is alkylated by ECH during synthesis and promotes the reaction of lignin contained in BL with ECH. Because of the lipophilic character of tertiary amines derived from aniline in this way, these compounds are co-extracted during the preparation of BLER and could also act as accelerator in polymerisation reactions involved in the formation of BLER-SA. Feltzin *et al.*^37^ found an activation energy of 27 kJ mol^−1^ for the polymerisation of a bisphenol-A diglycidyl ether with dimethyl benzyl amine and nadic acid anhydride. Given the structural similarity of the two systems, it seems not unlikely, that the activation energy determined here is influenced by aniline derivates remaining in BLER after synthesis. Surfaces of cured specimens were investigated using SE micrographs (Fig. 3f). The surface of cured BLER-SA appears smooth and without large pores. The material is homogeneous in general and no crystalline residues of the curing agent or inorganics were observed in the micrographs. No cracks were found in cured specimens that were cut to a size suitable for SEM. A gel phase could not be identified *via* SEM, indicating that gel is dissolved in the cured material.

**Fig. 4:**
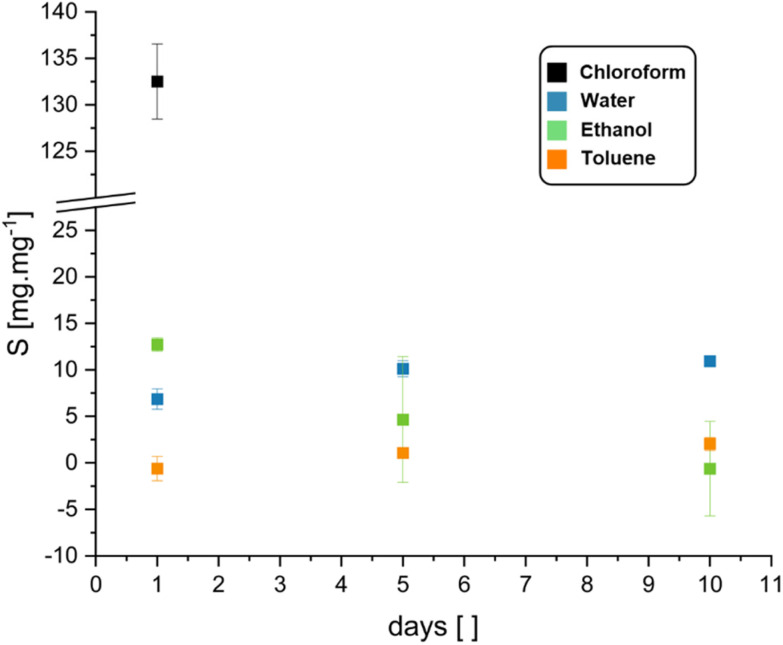
Swelling capacity (with standard deviation) of cured BLER-SA in water, ethanol, toluene, and chloroform over the course of 1, 5, and 10 days. Data for chloroform is only given for 1 day of submersion as after that time the specimens disintegrated due to severe swelling.

To investigate the stability of BLER-SA in solvents and the gel-content, we performed swelling and extraction tests. Water, ethanol, toluene and chloroform were used as solvents in the swelling tests. The gel-content was estimated by extraction with ethanol over ten days and was found to be 20 ± 6%. Ethanol was used as it visibly and measurably (loss of mass in swelling tests) extracted gel from used specimens without disintegrating them. The results of swelling tests *via* submersion in a given solvent are presented in Fig. 4. The most pronounced swelling of specimens was recorded in chloroform (132 ± 4% over one day), leading to specimen disintegration after one day of submersion. Specimens also swelled in ethanol, but as ethanol also extracted the gel-phase from the material it can only be reliably stated that the swelling occurs. The swelling data in ethanol, although given here, is likely not representative as negative swelling was recorded due to gel-extraction. Toluene and water reached a swelling equilibrium within 10 days of submersion. Toluene only led to low degrees of swelling of the material (2 ± 0.7% after 10 days), while water led to swelling of 11 ± 0.5% after 10 days without gel-extraction.

### Mechanical properties

**Fig. 5:**
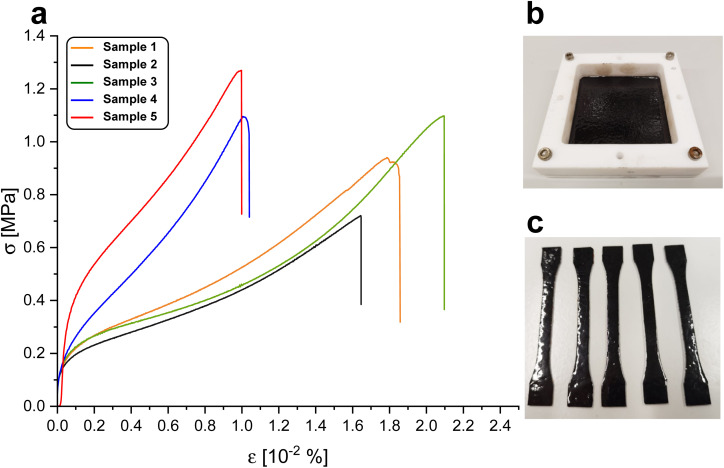
(a) Stress strain of five BLER-SA samples with an extension rate of 50 mm min^−1^. (b) Thin cured film of BLER-SA and (c) tensile samples stamped out from the film.

Tensile stress strain curves are depicted in Fig. 5a. Over five samples, the elongation at break was found to be 151 ± 49% of the initial length. The tensile strength over five samples was found to be 1.0 ± 0.20 MPa. The elastic modulus at 5% elongation was found to be 1.6 ± 1.4 MPa and decreased to a value of 0.44 ± 0.35 MPa at 50%. The relatively large differences in mechanical behaviour of the measured samples might arise from heat gradients in the oven during curing or post-treatment after curing. Gioia *et al.* reported values of a similar order of magnitude in lignin-based epoxy systems with *T*_g_ below 0 °C in mechanical tests using polyetheramine D2000 as hardener.^38^ Young's moduli ranging from 6 to 100 MPa and tensile strengths of 1.2 to 5 MPa were reported, while the elongation at break was lower than in the system reported here, ranging from 43–47%. In comparison with natural rubber, the system we report is inferior with respect to elongation at break and tensile strength by an order of magnitude.^39^ Wu *et al.* reported tensile strengths of silicon-/poly-butadiene rubber blends ranging from 2.3–2.5 MPa.^40^ Matsuura *et al.* reported a tensile strength below 2 MPa of a commercial silicon rubber (KE-78VBS, Shin-Etsu Chemical Co., Japan) prior to blending with linear low density polyethylene.^41^

**Fig. 6:**
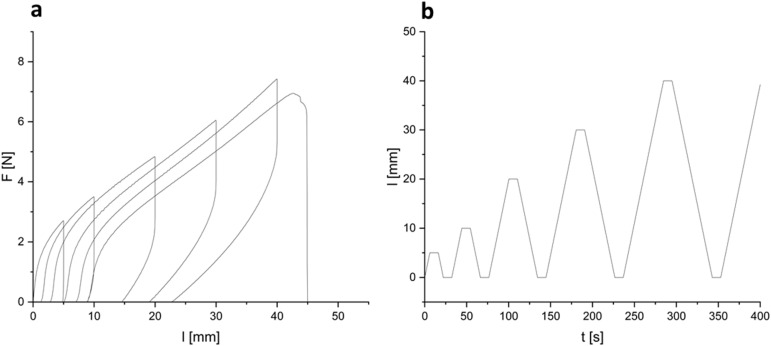
(a) Representative curves of load over elongation in cyclic tests of BLER-SA. (b) Elongation-over-time-profile in cyclic testing of BLER-SA.

To investigate the resistance of the material under cyclic stress (Fig. 6), samples were subjected to increasing extensions while monitoring load. Softening of the material was measured upon applied strain cycles. After a completed cycle, at the same elongation, a decrease in load was observed in the next cycle. A relatively stable ratio of 1.1 ± 0.1 N N^−1^ between the load of a previous cycle and the load of the following cycle was found. Since no filler was used, the weakening of the material was attributed to the breaking or reorientation of polymer chains within the material. Low strain recovery was observed even with a recovery time of 10 s, again indicating either damage upon induced strain or reorientation of polymer chains. The low elastic dimensional recovery/resilience of the material after unloading was attributed to a relatively high gel content and the damage to crosslinked (elastic) portions of the material due to applied stress. After having previously been subjected to stress, the material could not be stretched to an elongation of above 150%, but already failed at elongations below 100% (all curves are provided in ESI†). No significant decrease in Young's modulus could be observed over the cycles applied to a given specimen. A considerably lower maximum stress at break of 0.75 ± 0.13 MPa was recorded upon cyclic fatigue compared to a single elongation until break, further supporting considerable induction of damage upon elongation. Some specimens did not withstand six loading/unloading cycles. Shore hardness^42^ was measured to be 65 ± 1 after 3 s and 61 ± 2 after 15 s of measurement time. These values are in the range of lignin-reinforced rubbers tested by Setua *et al.* (40–85).^43^

### Flammability

**Fig. 7:**
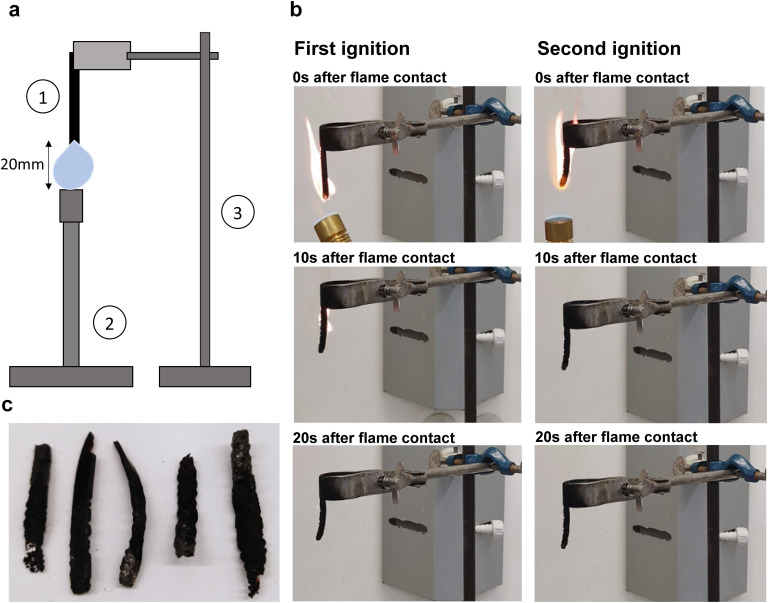
(a) Schematic drawing of vertical burning test setup. 1 indicates the elastomer sample, 2 indicates the Bunsen burner, and 3 indicates the stand with mounted clamp. (b) Representative images of samples during the first and second burning test phase. In the left column of pictures, the first ignition is depicted, in the right column the second ignition is depicted. (c) Tested samples after both tests.

To investigate the flame resistance of BLER-SA, we carried out vertical burning tests. The setup and impressions of the tests, as well as pictures of samples after the test, are shown in Fig. 7 and ESI.† Samples were clamped on a stand and ignited using a Bunsen burner. Five samples were characterised and all ignited within 10 s of flame contact. All samples burnt over the entire length of 50 mm during the first ignition. No dripping occurred, and the samples burnt with a sooty flame (photograph in ESI†), emitting a chemical smell. The mean first burning time over five samples was 17.6 s, with the lowest burning time being 10 s and the highest 29 s. No considerable second burning time was observed, with a mean burning time of 0.9 s and three out of five samples not igniting. The mean mass loss after the full test cycle was 56 wt%. Samples appeared bloated, brittle and fragile after burning. This behaviour is in accordance with TGA experiments, where similar values for mass loss in the temperature range of 200–300 °C were measured, and further relevant loss of mass only occurred above 550 °C. The material burns well and can sustain a flame. The occurrence of smoke and soot must also be assessed negatively. The short burning time after the second attempt to ignite the material is probably due to the fact that most of the material has already charred. A positive outcome in the vertical burning tests is the absence of melting or dripping of BLER-SA while burning. Given that the burn time was below 30 s and that no dripping occurred, BLER-SA has a flame-retardant classification of V-1. Dripping of burning polymer is very hazardous and can lead to fires propagating faster. Dripping is also associated with the formation of so-called pool fires (burning puddles of molten polymer) that can control the growth of a fire's propagation rate.^44^ On the other hand, the material seems to be particularly suitable for use as fuel after its service life as a polymer, meaning that it would not be alienated from the momentary use of lignin in industry (Fig. 7).

## Conclusion

We produced elastomeric thermosetting materials from untreated (raw) kraft black liquor using our previously published synthetic route^32^ leading to lignin-based epoxy resins. By using an anhydride hardener (succinic anhydride, SA) that has a flexible backbone and maintaining a gel-phase in the material, flexible thermosetting elastomers were produced. Our materials display, unfortunately, undesirable characteristics; loss of mechanical strength and elasticity upon stress/elongation, relatively high swelling in organic solvents paired with a low resistance to them due to the loss of a gel phase, and low resistance to ignition upon contact with an open flame. However, no meaningful loss in Young's modulus was found upon cyclic stress induction. In flame tests, the material ignited readily and burnt with a sooty flame. The absence of dribbling or melting during flame testing was a positive outcome of the latter. An imaginable application of this or similar materials derived from it could be the impregnation of, for example, cotton-based fabrics to produce clothing or furniture (similar to artificial leather).

## Author contributions

Philip Verdross conceptualised experimental and synthetic work, carried out all testing leading to data presented in the manuscript. Philip Verdross drafted the manuscript, made all figures/photographs and did all literature research presented. Alexander Bismarck acquired funding and gave input in regard of what testing should be done to characterise presented materials. Robert T. Woodward and Alexander Bismarck revised the manuscript together with Philip Verdross.

## Conflicts of interest

The authors Philip Verdross and Alexander Bismarck hold a patent together with the University of Vienna based on the production of BLER resin (WO 2024/062004).

## Supplementary Material

PY-015-D4PY00490F-s001
