# Supplementary material for: Flexing with lignin: lignin-based elastomers synthesised from untreated kraft black liquor
Source: Polym Chem. 2024 Jun 3;15(26):2687–97. doi: 10.1039/d4py00490f (PMC11217878; doi:10.1039/d4py00490f)
Supplement: PY-015-D4PY00490F-s001 [file PY-015-D4PY00490F-s001.pdf]

Electronic Supplementary Material (ESI) for Polymer Chemistry.
This journal is © The Royal Society of Chemistry 2024

Supplementary information

Flexing with lignin: Lignin-based elastomers synthesised from untreated kraft black liquor

Philip Verdross[a], Robert T[a]. Woodward*, Alexander Bismarck*;

[a]Polymer and Composite Engineering (PaCE) Group, Institute of Material Chemistry and Research, Faculty of Chemistry, University of Vienna, Wahringer ¨ Strasse, 42, 1090 Vienna, Austria

[b]Department of Chemical Engineering, Imperial College London, South Kensington Campus, London SW7 2AZ, United Kingdom

*Corresponding authors: robert.woodward@univie.ac.at; alexander.bismarck@univie.ac.at;

Pictures of samples used for tensile testing (cyclic and non-cyclic) and material in as produced in a Teflon mold

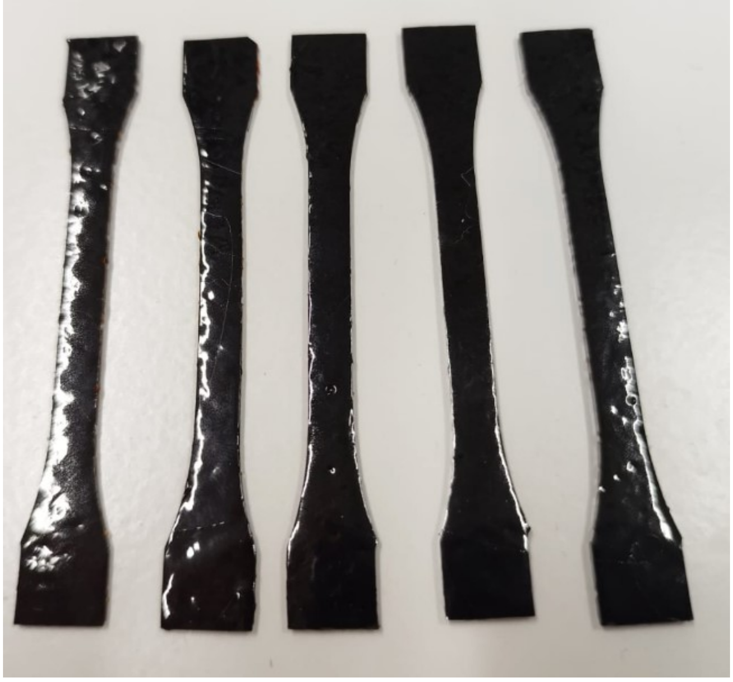

Figure 1: Example of test specimens used for cyclic mechanical tests and tensile testing

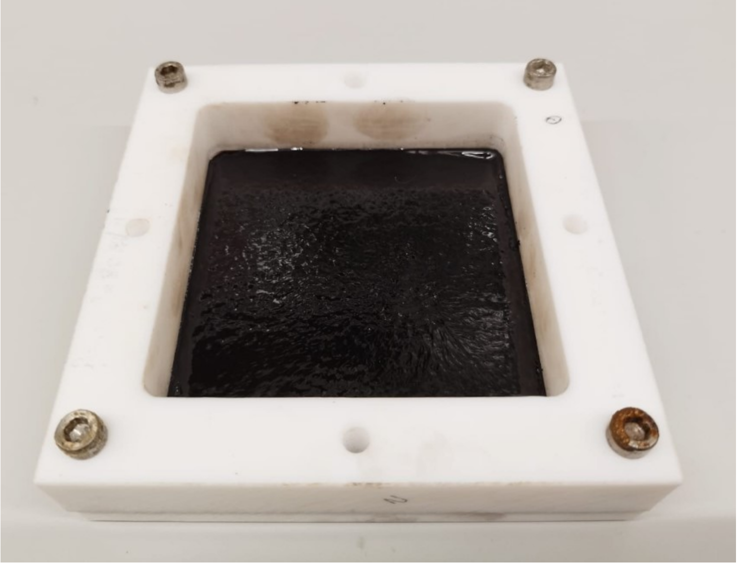

Figure 2: Mould with polymer used to produce films from which specimens for various tests have been cut/stamped out.

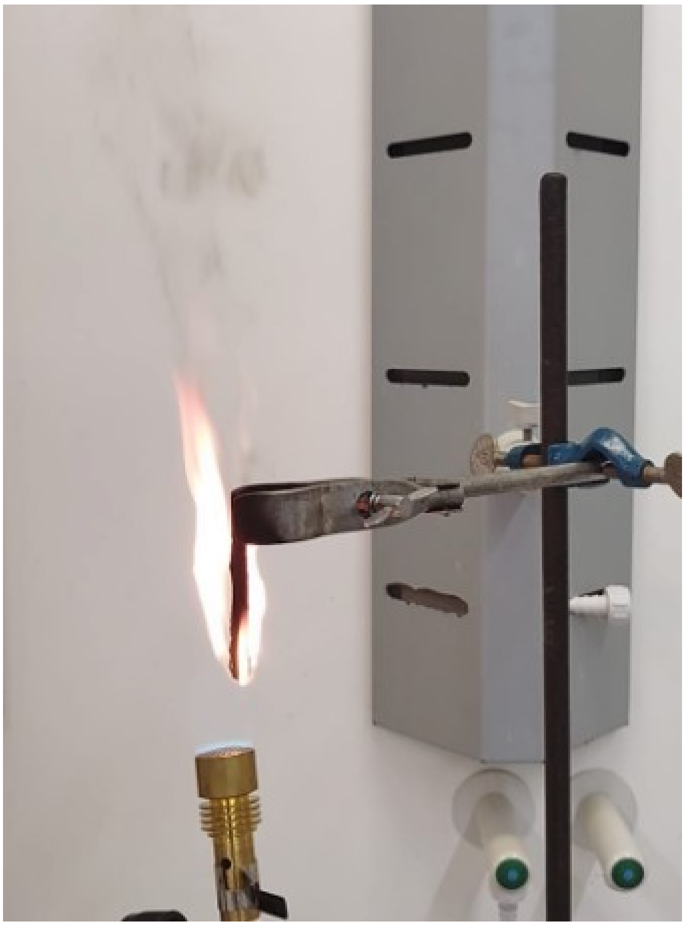

Figure 3: Impression of soot-evolution and flame during burn test.

Cyclic mechanical testing data

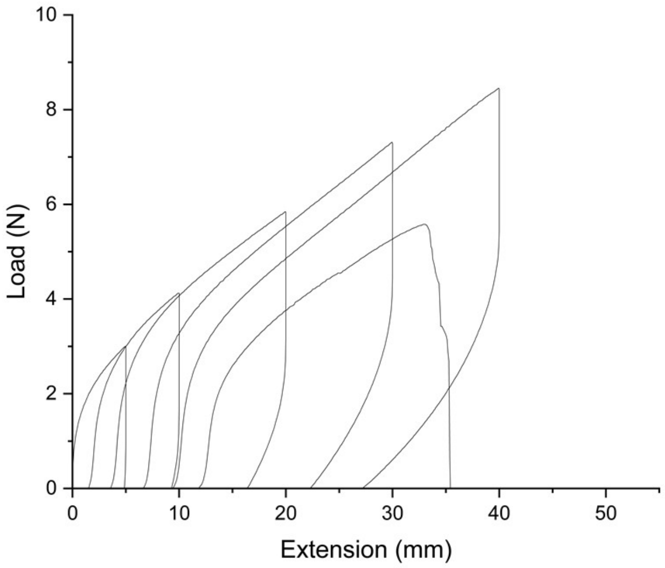

Figure 4: Curves of cyclic mechanical testing run.

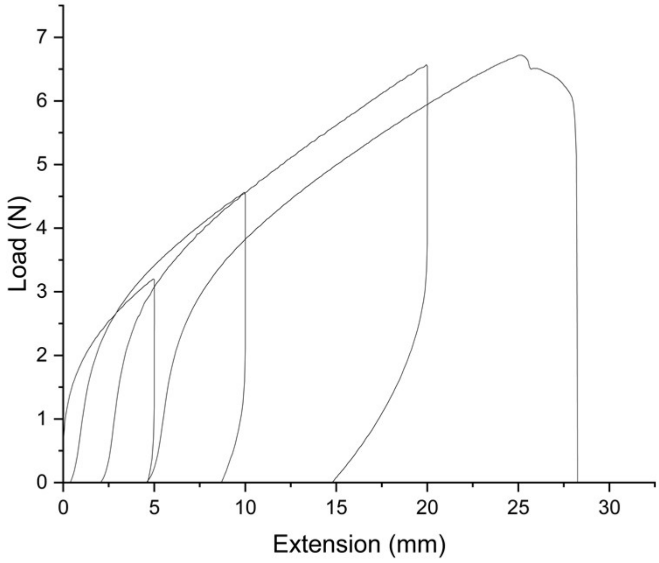

Figure 5: Curves of cyclic mechanical testing run.

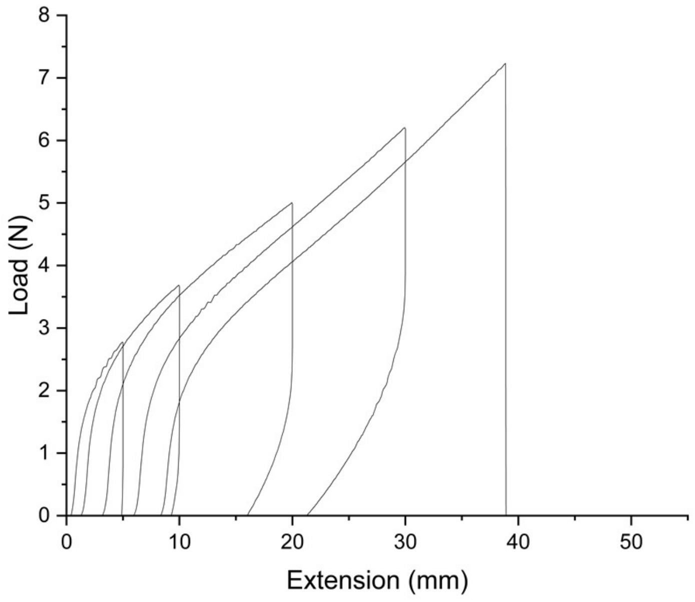

Figure 6: Curves of cyclic mechanical testing run.

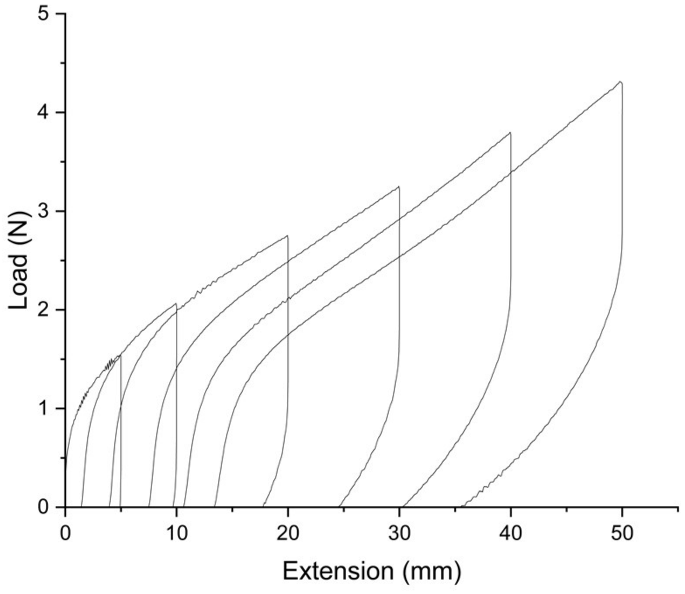

Figure 7: Curves of cyclic mechanical testing run.

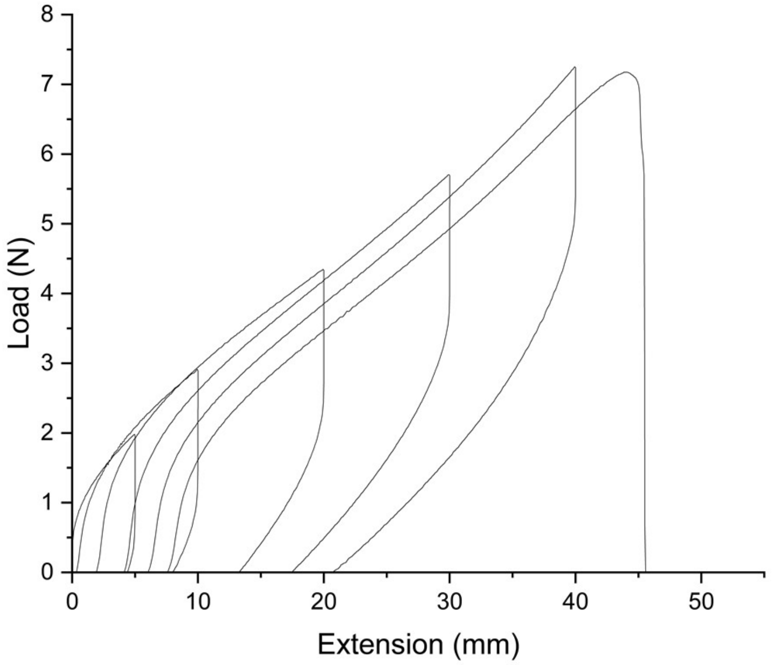

Figure 8: Curves of cyclic mechanical testing run.

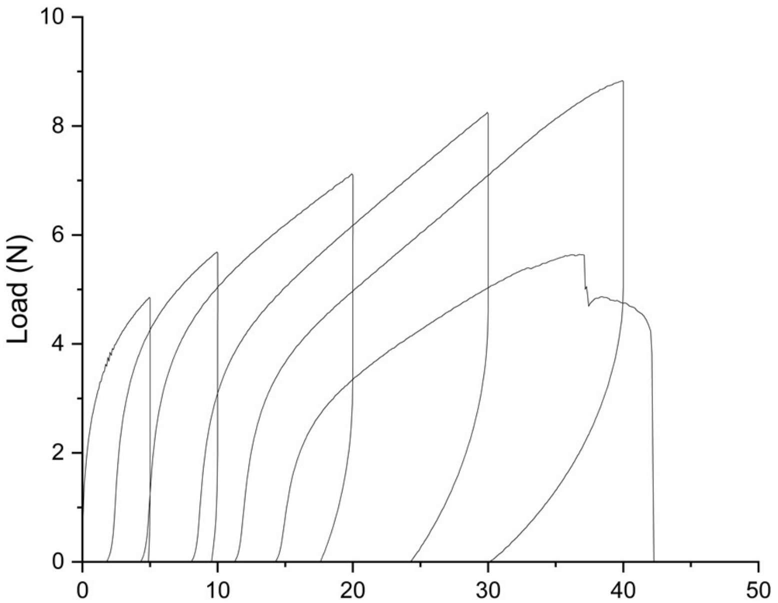

Figure 9: Curves of cyclic mechanical testing run.

Samples used for Shore tests

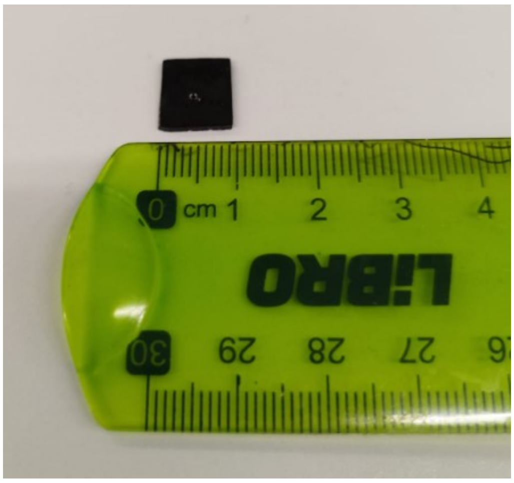

Figure 10: Typical sample used for shore hardness tests.

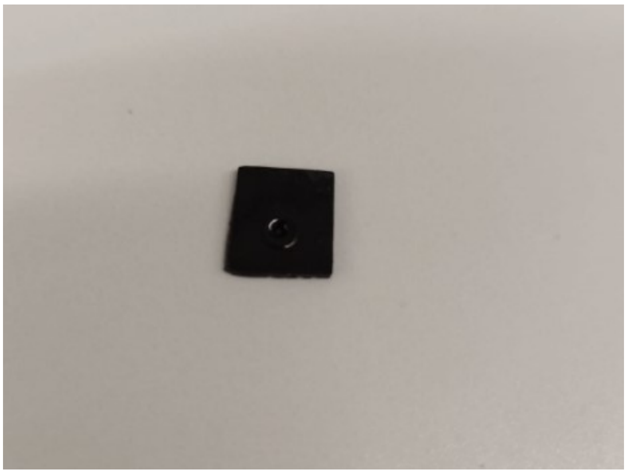

Figure 11: Sample immediately after shore hardness test.

FT-IR spectrum of recovered hemicellulose

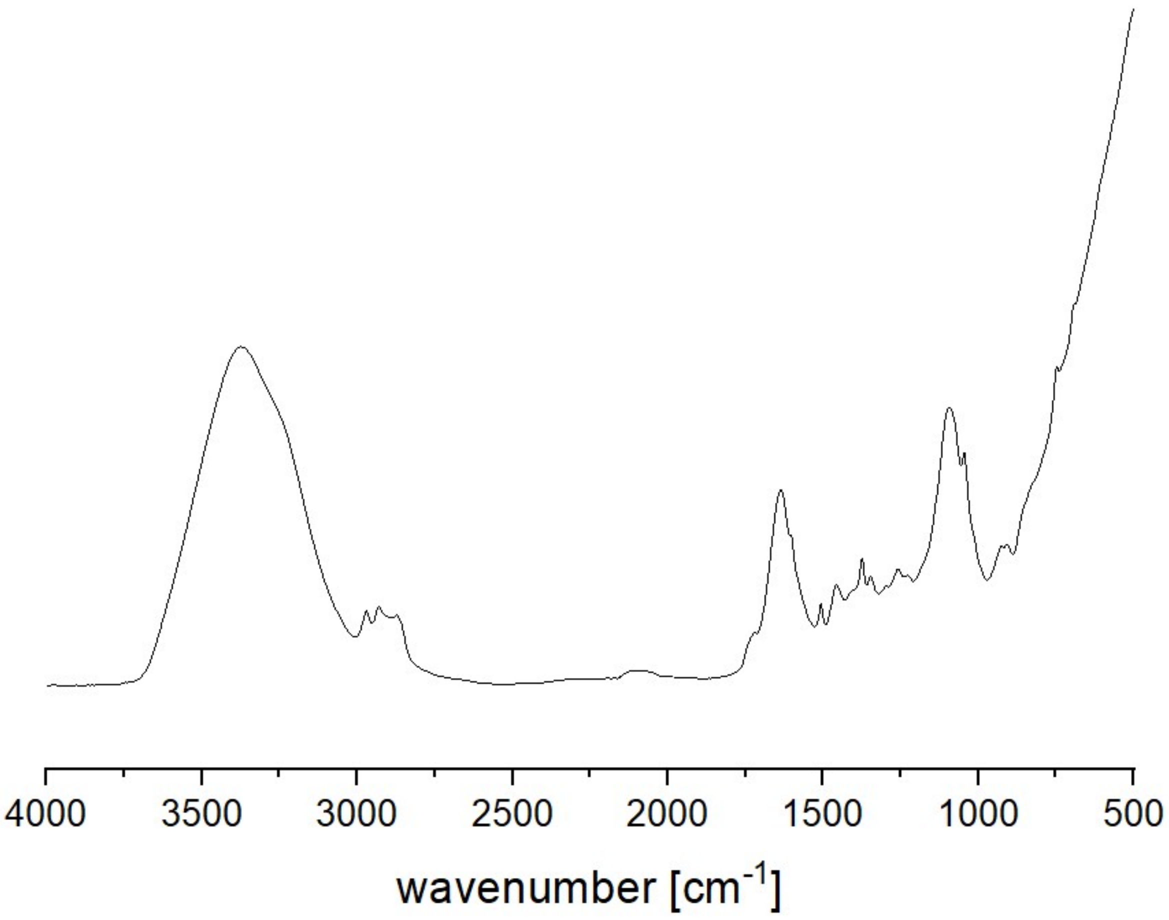

Figure 12: Infrared spectrum of dry hemicellulose recovered by acetone precipitation.
